# Conessine as a novel inhibitor of multidrug efflux pump systems in *Pseudomonas aeruginosa*

**DOI:** 10.1186/s12906-017-1913-y

**Published:** 2017-08-14

**Authors:** Thanyaluck Siriyong, Potjanee Srimanote, Sasitorn Chusri, Boon-ek Yingyongnarongkul, Channarong Suaisom, Varomyalin Tipmanee, Supayang Piyawan Voravuthikunchai

**Affiliations:** 1 Department of Microbiology and Excellence Research Laboratory on Natural Products, Faculty of Science and Natural Product Research Center of Excellence, Prince of Songkla University, Hat Yai, Songkhla, 90112 Thailand; 2 Graduate Program, Faculty of Allied Health Sciences, Thammasat University, Pathumthani, 12121 Thailand; 3 Faculty of Traditional Thai Medicine and Excellence Research Laboratory on Natural Products, Faculty of Science and Natural Product Research Center of Excellence, Prince of Songkla University, Hat Yai, Songkhla, 90112 Thailand; 4 Department of Chemistry and Center for Innovation in Chemistry, Faculty of Science, Ramkhamhaeng University, Bangkapi, Bangkok, 10240 Thailand; 5 Department of Biomedical Sciences, Faculty of Medicine, Prince of Songkla University, Hat Yai, Songkhla, 90112 Thailand

**Keywords:** Conessine, Efflux pump inhibitor, MexAB-OprM efflux system, *Pseudomonas aeruginosa*

## Abstract

**Background:**

*Holarrhena antidysenterica* has been employed as an ethnobotanical plant for the treatment of dysentery, diarrhoea, fever, and bacterial infections. Biological activities of the principle compound, conessine including anti-diarrhoea and anti-plasmodial effects were documented. Our previous study reported potency of *Holarrhena antidysenterica* extract and conessine as resistance modifying agents against extensively drug-resistant *Acinetobacter baumannii*. This study aimed to investigate (i) whether conessine, a steroidal alkaloid compound, could act as a resistance modifying agent against multidrug-resistant *Pseudomonas aeruginosa*, and (ii) whether MexAB-OprM efflux pump involved in the mechanism.

**Methods:**

Conessine combined with various antibiotics were determined for synergistic activity against *P. aeruginosa* PAO1 strain K767 (wild-type), K1455 (MexAB-OprM overexpressed), and K1523 (MexB deletion). H33342 accumulation assay was used to evaluate efflux pump inhibition while NPN uptake assay was assessed membrane permeabilization.

**Results:**

Conessine significantly reduced MICs of all antibiotics by at least 8-fold in MexAB-OprM overexpressed strain. The levels were comparable to those obtained in wild-type strain for cefotaxime, levofloxacin, and tetracycline. With erythromycin, novobiocin, and rifampicin, MICs were 4- to 8-fold less than MICs of the wild-type strain. Loss of MexAB-OprM due to deletion of *mexB* affected susceptibility to almost all antibiotics, except novobiocin. Synergistic activities between other antibiotics (except novobiocin) and conessine observed in MexB deletion strain suggested that conessine might inhibit other efflux systems present in *P. aeruginosa.* Inhibition of H33342 efflux in the tested strains clearly demonstrated that conessine inhibited MexAB-OprM pump. In contrast, the mode of action as a membrane permeabilizer was not observed after treatment with conessine as evidenced by no accumulation of 1-*N*-phenylnaphthylamine.

**Conclusions:**

The results suggested that conessine could be applied as a novel efflux pump inhibitor to restore antibiotic activity by inhibiting efflux pump systems in *P. aeruginosa*. The findings speculated that conessine may also have a potential to be active against homologous resistance–nodulation–division (RND) family in other Gram-negative pathogens.

## Background

*Pseudomonas aeruginosa* is an emerging global opportunistic multidrug-resistant (MDR) pathogen associated with high morbidity and mortality rates. The organism causes a number of infections such as pneumonia, urinary tract infection, and sepsis [1]. Broad spectrum antimicrobial resistance in MDR *P. aeruginosa* seriously limits effective therapeutic options. MDR phenotype can be mediated by a variety of resistance mechanisms including chromosomally encoded enzymes, expression of efflux pumps, and low membrane permeability. Various chromosomally encoded efflux systems and outer membrane porins have been identified as important contributors to resistance [1]. The most relevant multidrug efflux systems in MDR pathogens are members of resistance–nodulation–division (RND) family. A number of MDR RND efflux pumps have been characterized in clinical isolates of *P. aeruginosa*, namely MexAB-OprM, MexCD-OprJ, MexEF-OprN, and MexXY-OprM. Among these pumps, only MexAB-OprM is constitutively expressed at a level sufficient to confer intrinsic MDR in wild-type *P. aeruginosa* strains [2]. MexAB-OprM transports a number of antibiotics including fluoroquinolones, β-lactams, tetracycline, macrolides, chloramphenicol, novobiocin, trimethoprim, and sulphonamides [3]. Mutations in *nalB* or *mexR* resulted in overexpression of MexAB-OprM efflux pump [4].

Combination therapy may be beneficial for controlling MDR *P. aeruginosa* that could restore susceptibility to various antibiotics [5–7]. A number of potent efflux pump inhibitors including phenylalanyl arginyl β-naphthylamide (PAβN), carbonyl cyanide *m*-chlorophenylhydrazone (CCCP), quinoline derivatives, and 1-(1-Naphthylmethyl)-piperazine (NMP) have been reported to enhance antibiotic activity against antibiotic-resistant Gram-negative bacteria. In addition, various compounds such as PAβN, ethylenediaminetetraacetic acid (EDTA), and polymyxin B nonapeptide (PMBN) have been documented to permeabilize the bacterial outer membrane. However, none has reached potential clinical applications because of its toxicity [8].

A number of plant extracts and phytochemical products have demonstrated their potential as synergists or potentiators of other antibacterial agents [9]. Curcumin derived from *Curcuma longa* inhibited efflux pump systems in *P. aeruginosa*, resulting in restoring gentamicin and ciprofloxacin activity [10]. Extract from *Holarrhena antidysenterica* displayed resistance modifying ability to enhance novobiocin and rifampicin activity against *Acinetobacter baumannii* [11, 12]*.* It has been demonstrated that the extract potentiated the effect of antibiotics by acting as a permeabilizer [13]. Moreover, a recent study indicated that both *Holarrhena antidysenterica* extract and conessine, a steroidal alkaloid compound, could restore antibiotic activity due to interference with AdeIJK pump in *A. baumannii* [14]. Previous study documented that AdeIJK pump and MexAB-OprM pump are functionally equivalent pumps in both organisms [15].

*Holarrhena antidysenterica* belonging to family Apocynaceae has been employed as an ethnobotanical plant for the treatment of dysentery, diarrhoea, fever, and bacterial infections. Biological activities of the plant including antimalarial, anti-diabetic, anti-oxidant, anti-urolithic, anti-mutagenic, CNS-stimulating, angiotensin-converting-enzyme inhibitory, and acetylcholinesterase inhibitory activity were documented [16]. In contrast, anti-diarrhoea and anti-plasmodial effects of conessine were briefly mentioned [17].

This study aimed to investigate (i) whether conessine, a steroidal alkaloid compound, could act as a resistance modifying agent against multidrug-resistant *Pseudomonas aeruginosa*, and (ii) whether MexAB-OprM efflux pump is involved in the mechanism.

## Methods

### Bacterial strains

*P. aeruginosa* PAO1 strain K767 (wild-type), MexAB-OprM overexpressed strain K1455 (PAO1-*nalB*), and MexB deletion strain K1523 (PAO1-∆*mexB*) were generously provided by Professor Dr. R. Keith Poole, Queen’s University, Kingston, Ontario, Canada.

### Chemicals and media

Phenylalanine-arginine β-naphthylamide (PAβN), 1-*N*-phenylnaphthylamine (NPN), Hoechst 33,342 (H33342), conessine, and antibiotics were purchased from Sigma–Aldrich (St Louis, MO, USA). Dimethylsulfoxide (DMSO) and ethylenediaminetetraacetic acid (EDTA) were obtained from Merck (Merck, Germany).

Mueller-Hinton broth (MHB) and Tryptic soy agar (TSA) were purchased from Becton Dickinson Microbiology Systems (Sparks, MD, USA).

### Antibacterial activity assays

Minimum inhibitory concentration was tested by broth microdilution assay in accordance with the Clinical and Laboratory Standards Institute (CLSI) recommendation [18]. Antibiotics used in this study were selected based on substrate specificity of Ade efflux pump in *A. baumannii*: cefotaxime for AdeDE pump, novobiocin for AdeIJK pump, rifampicin for AdeDE and AdeIJK pump, erythromycin, levofloxacin, and tetracycline for AdeABC, AdeDE, and AdeIJK pump. In addition, cefotaxime, levofloxacin, novobiocin, and tetracycline have been reported as substrates for MexAB-OprM in *P. aeruginosa*. Stock solution of novobiocin (50 mg/L), rifampicin (1 mg/L), levofloxacin (18 mg/L), erythromycin (2 mg/L), cefotaxime (10 mg/L), and PAβN (10 mg/L) were prepared in sterile deionized water. Tetracycline (4 mg/L) and conessine (1 mg/L) were dissolved in 95% ethanol and 100%DMSO, respectively. Serial dilutions of conessine, PAβN, and antibiotics were prepared in MHB. In order to investigate the effect of each agent, 100 μL bacterial culture (1 × 10^6^ cfu/mL) was mixed with 100 μL each conessine, PAβN, or antibiotics. Synergistic effects of conessine (20 mg/L) or PAβN (25 mg/L) and antibiotics were assessed using checkerboard assay by adding 100 μL culture into a well containing 50 μL conessine or PAβN and 50 μL antibiotics. DMSO at a final concentration of 1% used as a negative control and PAβN, an efflux pump inhibitor was used as a positive control. Plates were then read after 18 h of incubation at 37 °C. Each assay with three biological triplicates was repeated at least twice. A 4-fold or greater reduction in MIC values after addition of conessine or PAβN was considered significant. Fractional inhibitory concentration index (FICI) value was calculated for each combination according to the following formula [19]: FICI = (MIC of efflux pump inhibitors in combination/MIC of efflux pump inhibitors alone) + (MIC of antibiotics in combination/MIC of antibiotics alone). Synergy, additivity, and antagonism were defined as FICI <1, =1, and >1, respectively.

### H33342 accumulation assay

H33342 accumulation assay was performed to evaluate the effect of efflux pump inhibitors on the activity of MexAB-OprM efflux pump [20]. Briefly, overnight bacterial cultures were inoculated into MHB and rotated at 250 rpm at 37 °C for 4–5 h. Bacterial cells were harvested by centrifugation (3000 rpm for 15 min) and the cells were washed with phosphatebuffered saline containing 1 mM MgSO_4_ and 20 mM glucose. After centrifugation, the pellets were resuspended in the same buffer and OD_600_ of each suspension was adjusted to 0.4. An aliquot of 100 μL of the bacterial suspension was added into a well in black microtiter plate containing each of 50 μL conessine (20 mg/L) or an efflux pump inhibitor, PAβN (25 mg/L).

The final concentration of DMSO in all assays was ≤1%. Plates were incubated at 37 °C for 15 min and 50 μL H33342 (2.5 μM) was added to each assay well. Fluorescence (excitation 355 nm, emission 460 nm) was measured at 37 °C every 2.30 min for 1 h using a Varioskan Flash spectral scanning multimode reader. Each assay was repeated at least twice. Differences in accumulation in the presence of efflux pump inhibitors compared with the absence of efflux pump inhibitors were analysed for statistical significance using Student’s *t*-test. *P* value ≤0.05 was considered significant.

### NPN uptake assay

Ability of conessine to permeabilize *P. aeruginosa* outer membrane was assessed by NPN uptake assay [21]. NPN, an uncharged lipophilic molecule, fluoresces weakly in aqueous environments but becomes strongly fluorescent in nonpolar environments such as cell membranes. Briefly, overnight bacterial cultures were inoculated into MHB and rotated at 250 rpm at 37 °C for 4–5 h. Bacterial cells were harvested at 3000 rpm for 15 min, washed with 100 mM NaCl and 50 mM sodium phosphate buffer (pH 7.0), and resuspended in the same buffer at A 600 = 0.1 in the presence of 0.05% of glucose. An aliquot of 100 μL of the bacterial suspension was added into a well in black microtiter plate containing each of 50 μL conessine (20 mg/L) or EDTA (100 μM) as a permeabilizer followed by adding 50 μL of NPN (40 μM). The final concentration of DMSO in all assays was ≤1%. NPN fluorescence intensity (excitation 322 nm, emission 424 nm) was monitored at 37 °C after 2.30 min for 1 h using a Varioskan Flash spectral scanning multimode reader (Thermo Fisher Scientific, Finland). Each assay was repeated at least twice. Differences in accumulation in the presence of efflux pump inhibitors compared with the absence of efflux pump inhibitors were analysed for statistical significance using Student’s *t*-test. *P* value ≤0.05 was considered significant.

## Results

### Intrinsic antibacterial activity of conessine, PAβN, and antibiotics

MICs of conessine and PAβN for *P. aeruginosa* wild-type strain K767 (PAO1), MexAB-OprM overexpressed strain K1455 (PAO1-*nalB*), and MexB deletion strain K1523 (PAO1-∆*mexB*) were determined. Intrinsic MICs of conessine in all strains were 40 mg/L while MICs of PAβN were between 512 and 1024 mg/L. At a concentration required to reduce MICs of antibiotics by at least 4-fold in *P. aeruginosa* strains*,* conessine (20 mg/L) and PAβN (25 mg/L) alone did not affect growth rate in all strains (data not present).

Susceptibility of *P. aeruginosa* strains to a range of antibiotics is shown in Table 1. Overexpression of MexAB-OprM conferred resistance to cefotaxime, erythromycin, levofloxacin, novobiocin, and tetracycline, except rifampicin. The experiments supported earlier report that resistance to rifampicin may involve mutations in *rpoB* gene rather than as a simple function of expression of efflux systems [22]. A mutant strain with MexB deletion displayed susceptibility to cefotaxime, levofloxacin, and novobiocin. The findings indicate that extrusion of cefotaxime, levofloxacin, and novobiocin was mainly specific to MexAB-OprM efflux system. On the other hand, resistance to erythromycin, rifampicin, and tetracycline may involve other efflux pump systems. Together, the results are consistent with previous studies on substrate specificity of Mex efflux systems in *P. aeruginosa* [23, 24].

**Table 1:** Modulation of antibiotic resistance in *Pseudomonas aeruginosa* by conessine

Bacterial strain	Antibiotic	Antibiotic Minimum inhibitory concentration (mg/L) with			Fractional inhibitory concentration index^b^	
		No EPI^a^	Conessine (20 mg/L)	PAβN (25 mg/L)	Conessine	PAβN
K767 (PAO1)	Cefotaxime	8	1	4	0.63	0.52
	Erythromycin	128	**16**	64	0.63	0.52
	Levofloxacin	0.25	**0.03**	**0.03**	0.63	0.15
	Novobiocin	1024	**128**	**128**	0.63	0.15
	Rifampicin	16	**2**	**2**	0.63	0.15
	Tetracycline	8	**1**	4	0.63	0.52
K1455 (PAO1-*nalB*)	Cefotaxime	64	**8**	64	0.63	1.02
	Erythromycin	256	**32**	**64**	0.63	0.27
	Levofloxacin	2	**0.25**	**0.25**	0.63	0.15
	Novobiocin	>2048	**256**	**256**	0.63	0.15
	Rifampicin	16	**2**	**2**	0.63	0.15
	Tetracycline	64	**8**	64	0.63	1.02
K1523 (PAO1-*∆mexB*)	Cefotaxime	1	**0.13**	**0.13**	0.63	0.17
	Erythromycin	128	**16**	64	0.63	0.55
	Levofloxacin	0.06	**0.01**	**0.01**	0.63	0.17
	Novobiocin	64	64	64	1.50	1.05
	Rifampicin	16	**2**	**2**	0.63	0.17
	Tetracycline	8	**1**	**2**	0.63	0.30

A reduction of at least 4-fold is indicated in bold

^a^EPI, Efflux pump inhibitor

^b^Synergy, <1; additivity, =1; antagonism >1

### Efflux pump inhibitor activity of conessine in *P. aeruginosa*

To verify that a mechanism by which conessine potentiate antibacterial activity was through inhibition of MexAB-OprM efflux, this study determined whether conessine enhanced antibacterial activity against wild-type, MexAB-OprM overexpressed, and MexB deletion strain. As shown in Table 1, antibiotic spectrum was affected by the addition of conessine and PAβN. Conessine significantly reduced MICs of all antibiotics by at least 8-fold in wild-type and MexAB-OprM overexpressed strain. In the overexpressed strain, the levels were comparable to those obtained in wild-type strain for cefotaxime, levofloxacin, and tetracycline. Interestingly, with erythromycin, novobiocin, and rifampicin, MICs were 4- to 8-fold less than those in the wild-type strain. With mexB deletion strain, conessine increased its susceptibility to almost all antibiotics, except novobiocin. Previous studies documented that other efflux pumps such as MexAB-OprM and MexCD-OprJ could export novobiocin [23, 24]. However, no synergistic effect was observed in MexB deletion strain, indicating that conessine may inhibit only MexAB-OprM pump to restore novobiocin activity. The results clearly demonstrated that conessine could be an inhibitor of MexAB-OprM efflux pump. Synergistic activity between other antibiotics (except novobiocin) and conessine observed in MexB deletion strain suggested that conessine might inhibit other efflux systems present in *P. aeruginosa*. Conessine increased susceptibility to rifampicin in all strains when this agent was not exported by MexAB-OprM efflux pump, indicating that conessine enhanced antibiotic susceptibility in MexAB-OprM overexpressed strain independent of an impact on MexAB-OprM. Restoration of rifampicin efficacy by conessine might result in increase in antibiotic susceptibility. In addition, conessine has the same 8-fold reduction impact on antibiotic resistance in the wild-type, mexB deletion, and MexAB-OprM overexpressed strain, suggesting that conessine may act on other intrinsic resistance determinants.

A well-known inhibitor of RND efflux systems, PAβN [5], increased susceptibility to all antibiotics in the wild-type strain whereas in MexAB-OprM overexpressed strain, the inhibitor could not reduce MICs of tetracycline and cefotaxime. In MexB deletion strain, MICs of all antibiotics except novobiocin were affected by PAβN. The results are consistent with previous reports indicating that PAβN inhibited MexAB-OprM pump as well as other Mex efflux systems in *P. aeruginosa* such as MexCD-OprJ and MexEF-OprN [5, 25]*.* Remarkably, conessine could decrease MICs of levofloxacin, novobiocin, and rifampicin in all strains to the levels comparable to those when combined with PAβN. With cefotaxime, erythromycin, and tetracycline, conessine could lower the MICs in all the strains better than PAβN. The findings clearly demonstrated that conessine could reduce MIC against *P. aeruginosa* 2- to 8-fold lower magnitude in the tested strains, compared with PAβN.

### Inhibition of efflux systems

**Fig. 1:**
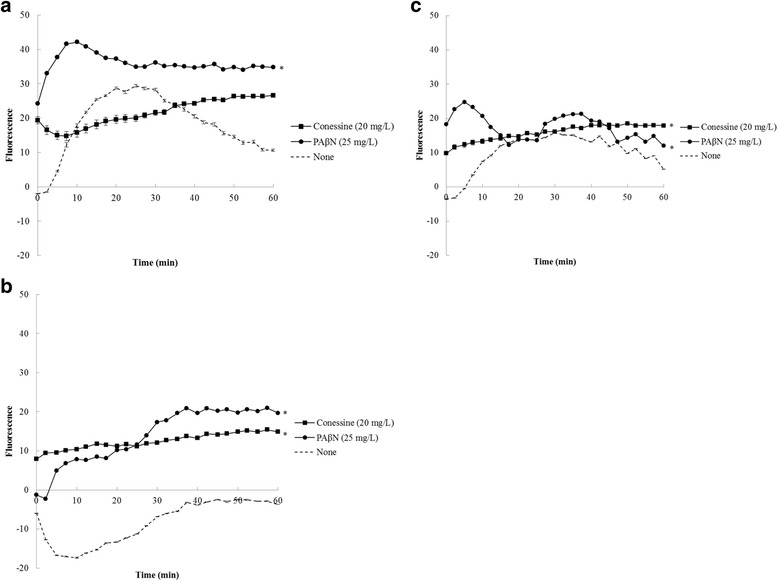
Intracellular concentration of Hoechst 33,342 in the presence of conessine (20 mg/L) and phenylalanine-arginine β-naphthylamide (PAβN) (25 mg/L) in *Pseudomonas aeruginosa* K767 (PAO1) (**a**), *P. aeruginosa* K1455 (PAO1-*nalB*) (**b**), and *P. aeruginosa* K1523 (PAO1-∆*mexB*) (**c**). Data were shown as average of two independent experiments. Error bars displayed ±SEM and * indicated significant (*P* value ≤0.05)

H33342 accumulation assay was used to confirm that conessine directly inhibited efflux pump systems in *P. aeruginosa* [20]*.* Conessine at 20 mg/L demonstrated an increase in H33342 accumulation in a wild-type strain (Fig. 1a). However, the effect of conessine was less than PAβN. A similar pattern was observed from an overexpressed strain (Fig. 1b). In a pump-deficient strain (Fig. 1c), conessine could elevate the level of H33342 accumulation to the level comparable to those when combined with PAβN. The findings suggested that conessine inhibited MexAB-OprM efflux pump and other efflux pump systems in *P. aeruginosa*.

### Interaction with outer membrane of *P. aeruginosa*

**Fig. 2:**
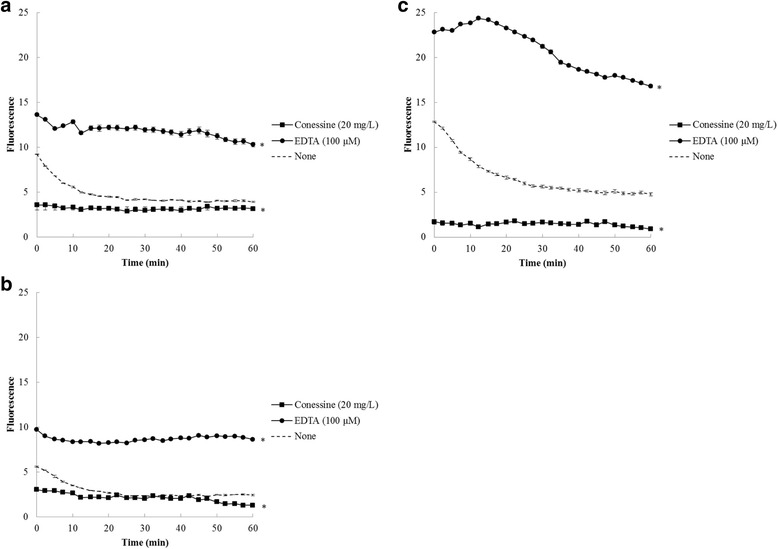
Intracellular concentration of 1-*N*-phenylnaphthylamine in the presence of conessine (20 mg/L) and ethylenediaminetetraacetic acid (EDTA) (100 μM) in *Pseudomonas aeruginosa* K767 (PAO1) (**a**), *P. aeruginosa* K1455 (PAO1-*nalB*) (**b**), and *P. aeruginosa* K1523 (PAO1-∆*mexB*) (**c**). Data were shown as average of two independent experiments. Error bars displayed ±SEM and * indicated significant (*P* value ≤0.05)

Ability of conessine to permeabilize outer membrane of *P. aeruginosa* was determined with intact cells by NPN uptake assay [21]. As shown in Fig. 2, conessine (20 mg/L) did not weaken outer membrane of all *P. aeruginosa* strains as evidenced by no increase in NPN uptake. Conversely, a positive control EDTA (100 μM) promoted NPN uptake across outer membrane of all tested strains as indicated by a significant increase in NPN uptake. The findings suggested that conessine has no effect on outer membrane permeability. Different *P. aeruginosa* genotypes exhibited different level of fluorescence especially in MexB deletion strain which demonstrated the highest level of fluorescence. Regarding MexAB-OprM overexpressed strain, the level of fluorescence was lower than in wild-type due to the efflux pump activity of MexAB-OprM. Conessine did not increase NPN uptake in all the tested strains, thus fluorescence level after the bacterial cells treated with conessine were the same. The level of fluorescence in the presence of conessine did not lower than the control, in particular for a pump-deficient strain (Fig. 2c) but because of the increase in fluorescence in the deletion strain.

## Discussion

Inhibition of MexAB-OprM efflux pump appears to be an attractive approach to restore efficacy of antibiotics that were substrates of this pump. Herein, we describe efficacy of conessine, an inhibitor of RND class MexAB-OprM efflux pump, which is the major efflux pump and plays a vital role in MDR phenotype in *P. aeruginosa*. The present study demonstrated that conessine displayed characteristics of an efflux pump inhibitor as previously documented by Lomovskaya et al. [5].

A mechanism of efflux pump inhibition by conessine was possibly through competitive inhibition and/or blockage of access to the substrate binding site of MexB. In comparison with MexB-specific PAβN, the compounds might interact with “G-loop” or “switch loop”, which separates the distal and the proximal binding sites. G-loop has been proposed to be involved in movement of substrates from the proximal to the distal site. Therefore, efflux pump inhibitors inhibited MexB extrusion of various substrates through binding to G-loop [26]. Differences in spectrum of antibiotics enhanced by conessine versus PAβN suggested that conessine may bind to a different site in MexB binding pocket.

## Conclusion

Conessine potentiated antibiotic activity by inhibiting MexAB-OprM efflux pump in *P. aeruginosa*. The findings revealed that a resistance modifying agent, conessine may be employed as a novel inhibitor for the alternative therapy against multidrug-resistant *P. aeruginosa*.

## Abbreviations

CCCP: Carbonyl cyanide m-chlorophenylhydrazone
CLSI: Clinical and Laboratory Standards Institute
DMSO: Dimethylsulfoxide
EDTA: Ethylenediaminetetraacetic acid
EPI: Efflux pump inhibitor
FICI: Fractional inhibitory concentration index
H33342: Hoechst 33342
MDR: Multidrug resistant
MHB: Mueller hinton broth
MHB: Mueller-Hinton broth
MIC: Minimal inhibitory concentration
NMP: 1-(1-Naphthylmethyl)-piperazine
NPN: 1-*N*-phenylnaphthylamine
PAβN: Phenylalanyl arginyl β-naphthylamide
PMBN: Polymyxin B nonapeptide
RND: Resistance–nodulation–division
TSB: Tryptic soy broth
